# Decreased cold‐sensing function of the transient receptor potential channel TRPM8 from tailed amphibians

**DOI:** 10.1002/2211-5463.70227

**Published:** 2026-03-25

**Authors:** Tadahiro Sawao, Daiki Kobayashi, Shogo Hori, Osamu Saitoh

**Affiliations:** ^1^ Graduate School of Biosciences Nagahama Institute of Bio‐Science and Technology Shiga Japan; ^2^ Department of Animal Bio‐Science Faculty of Bio‐Science, Nagahama Institute of Bio‐Science and Technology Shiga Japan; ^3^ Genome Editing Research Institute, Nagahama Institute of Bio‐Science and Technology Shiga Japan

**Keywords:** amphibian, channel, neuron, thermosensing, transient receptor potential

## Abstract

Sensing environmental temperature is a complex process involving an array of channels expressed by nociceptive sensory neurons but is critical to allow animals to move away from suboptimal environments and find environments with ideal temperatures. Focusing on one such channel (the transient receptor potential channel TRPM8), we have investigated the function of this channel across four tailed amphibians, which prefer cooler environments, studied behavioral responses to cold temperature, and characterized their TRPM8 function. Unlike frogs, which are known to prefer warmer climates, we observed that tailed amphibians do not show escaping behavior to avoid cold temperatures. Examination of the temperature‐response profiles of TRPM8 channels across these species demonstrated that tailed amphibian TRPM8 commonly has diminished cold sensitivity compared to frog TRPM8. Furthermore, we identify that eight amino acids at the N terminus of the TRPM8 protein determine the low‐temperature sensitivity of the channel. While further work is necessary to understand how these amino acids regulate the cold‐induced opening of the TRPM8 channel, these results represent key findings highlighting the role of TRPM8 in environmental temperature sensing.

## Graphical abstract

Despite frogs avoiding low temperatures, examination of four salamander species revealed that none avoided cold and all possessed cold tolerance. Functional analysis of TRPM8, a cold sensor, showed that all salamander TRPM8s had lost their cold sensitivity. Mutations causing loss of cold sensitivity were found in a common region of salamander TRPM8s, suggesting that acquiring these mutations enabled cold adaptation.

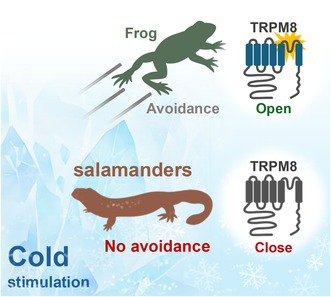

**Abbreviations**
AA: amino acid
ANK: Ankyrin Repeat
Ax: axolotl
IR Newt: Iberian ribbed newt
JC Sal: Japanese clawed salamander
MHR: melastatin homology region
TM: transmembrane
TRP: transient receptor potential
Y Sal: Yamato Salamander

The thermosensation mechanism is necessary for animals to survive in various thermal environments. Animals use subsets of transient receptor potential (TRP) channels (thermoTRPs) as main thermal sensors. ThermoTRPs in mammals are TRPA (TRPA1), TRPV (TRPV1‐TRPV4), and TRPM (TRPM2, TRPM4, TRPM5, and TRPM8) subfamilies, and each thermoTRP is activated at a specific and different temperature range [1]. Nociceptive sensory neurons express primarily TRPV1, TRPA1, TRPM8, and TRPC5, which act as sensors of nociceptive temperature and chemical stimuli [1, 2, 3, 4]. The temperature that activates each thermoTRP in rodents and humans has been well‐investigated and determined [1]. But, since animals live and thrive throughout the planet from the frigid polar region to the scorching desert, they cannot utilize the TPR sensors with the same thermal‐sensing characters and a proper sense of temperature must have been provided by obtaining the functional changes of the thermoTRP molecules.

Therefore, we have started the studies on thermoTRP channels from members of the order Urodela, which are tailed amphibians. Since most of these animals prefer cooler environments, we hypothesized that these animals express unique thermoTRPs contributing to their habitat selection. First, by electrophysiological analysis, we examined the properties of TRPV1s from four tailed amphibians [Axolotl (Ax, *Ambystoma mexicanum*), Iberian ribbed newt (IR Newt, *Pleurodeles waltl*), Yamato salamander (Y Sal, *Hynobius vandenburghi*), and Japanese clawed salamander (JC Sal, *Onychodactylus japonicus*)]. While many TRPV1s are known to be activated above about 40 °C [rat: [5], human: [6], chick: [7], frogs (*X. laevis* and *X. tropicalis*): [8]], our results showed that the TRPV1 channels from all four tailed amphibians are heat‐activated at lower temperatures between 28 °C and 34 °C. Studies on characterizing chimera and point mutants of the TRPV1s from axolotls and rats revealed that two amino acid residues in the Ankyrin Repeat 1 (ANK1) region of the N‐terminal domain are required to determine the heat‐activation threshold of TRPV1. These are Q128 and L154 in axTRPV1 and R114 and K140 at the corresponding positions in rat TRPV1. Further studies indicated that two amino acids at the same positions of other urodelan TRPV1s play an important role in reducing their heat‐activation threshold. Thus, we found that tailed amphibians express TRPV1s with a low‐temperature threshold for activation, which may have occurred by the substitution of two amino acids at ANK1 to contribute to cool‐habitat selection [9].

In addition to heat sensation, cold sensation is also important to adapt to environmental temperature. The free endings of sensory neurons express transduction and voltage‐dependent ion channels forming their electrical response induced by cold stimulation. TRPM8 is known to be a key molecule to trigger this cold transduction [10, 11, 12, 13, 14]. While rat TRPM8 is activated by cold stimulation from 24 °C, cold stimulation is tuned during evolution in several tested vertebrate species [15, 16, 17, 18, 19]. Here, we focused on TRPM8 from tailed amphibians, which prefer cooler environments. We first isolated TRPM8 cDNA from Y Sal. Y Sal is a lowland and still‐water breeder distributed in the Kinki and Chubu regions of Japan [20, 21]. In winter, its breeding behavior begins in water at nearly 4 °C. It was considered that this animal must have the TRPM8 with unique property. Further, we compared the cold‐sensing function of four tailed amphibians. Then, we approached the mechanism regulating the features of tailed amphibian TRPM8.

## Materials and methods

### Experimental animals

All of the animal experiments described below were approved by the Animal Experiment Committee of Nagahama Institute of Bio‐Science and Technology (#078, #114) and were performed based on their guidelines. Axolotl (Ax, *Ambystoma mexicanum*), Iberian ribbed newt (IR Newt, *Pleurodeles waltl*), Yamato salamander (Y Sal, *Hynobius vandenburghi*), and western clawed frog (WC frog, *Xenopus tropicalis*) were kept at 20–25 °C. Japanese clawed salamander (JC Sal, *Onychodactylus japonicus*) inhabits relatively high‐altitude mountain spring water, so it was kept in a refrigerator at 4–7 °C. For electrophysiological experiments, African clawed frog (*Xenopus laevis*) was kept at 18 °C and oocytes were utilized as described previously [22].

### Behavioral experiments

For tailed amphibians, we used animals aged 2–4 years, measuring approximately 7–10 cm. As a control, we used WC frogs about 5 cm long at ages of 2–4 years. Sex determination of tailed amphibians was not possible, because it is difficult to do so until the breeding season. The frogs were also used in the experiment without sexing. For behavioral experiments of Ax, IR newt, and WC frog, five animals were utilized. In case of Y Sal experiment, we used three animals.

Each amphibian was placed into a plastic round case beaker with chlorine‐free tap water at room (20 °C or 25 °C) or cold (6 °C or 8 °C) temperatures. We monitored the locomotor activity of the animals using a video camera. After placing them into the tap water, cold‐induced movements per 1 s were measured over 0–60 s and the mean movement (cm) per 1 min was calculated.

### Cloning of TRPM8 cDNAs from tailed amphibians and chimera construction

Based on axolotl (Ax, *Ambystoma mexicanum*) EST sequences (NT_01000039893.1, NT_0100002750284.1, https://www.axolotl‐omics.org/), a cDNA fragment of TRPM8 was PCR‐amplified using the following primers from the brain of axolotl and its sequence was determined.

3′F: CCTCCACCATCAAGGAGAAGTTG

5′R: TCCAACTGGTTCCACTCCAGGAG

3′Nested_F: CCGGCTATCCGAGGAGGAAAC

5′Nested_R: TTGTTCATTGGTACTGAATGCTTTG

In order to amplify cDNA fragments of other tailed amphibian TRPM8s, the following primers were generated based on an EST sequence (M0093872_PLEWA04, M0093872_PLEWA02, M0093872_PLEWA02, Trinity_Pwal_v2.fasta.gz, https://www.nibb.ac.jp/imori/main/) of Iberian ribbed newt (IR Newt, *Pleurodeles waltl*).

3′F: AGAACTGGAATGCCCAACTGAAG

5′R: CTGCAGAACTGCCCAGATGAAG

3′Nested_F: GGAATCAGTTGGATCTGGCCAG

5′Nested_R: GGTGTCTTGTGATCGGAGATGTCTC

The nucleotide sequences of amplified DNA fragments of TRPM8 cDNA from IR newt, Yamato salamander (Y Sal, *Hynobius vandenburghi*), and Japanese clawed salamander (JC Sal, *Onychodactylus japonicus*) were determined.

Once the expected partial sequence of each TRPM8 cDNA was obtained, the sequence information of the full‐length cDNA was determined by the Rapid Amplification of cDNA Ends (RACE) method using GeneRacer™ kit (ThermoFisher Scientific, Waltham, MA). To isolate full‐length cDNAs of Ax and IR newt TRPM8 using the RACE method, we used the same sets of primers used for PCR amplification of the first partial cDNA fragments of TRPM8. On the other hand, to isolate Y Sal and JC Sal TRPM8 cDNAs using the RACE method, the following primers were generated based on the determined sequence of each partial TRPM8 fragment and utilized.

Y_Sal

3′F: TCTTCACCAATGACAGACGCTGGGAG

5′R: CAAGCCGTCCTTCATGCTTCCATC

3′Nested_F: CTGACTTGGAGGACGCCATGTTCTTG

5′Nested_R: GGAAGTTGGTGATCATCTTCCAGACGAAG

JC_Sal

3′F: GAGTCCGCTGACTTGGAGGATGCTATG

5′R: GACGTGTCCTTTCCGACAATTTGCAC

3′Nested_F: CTGGTAAAGGACCAGCCGAAATTCGTC

5′Nested_R: TCTTTGGAAGTTGGTGACCATCTTCCAG

The partial genomic sequence encoding Y Sal TRPM8 was determined. We amplified genomic sequence surrounding regions where differences between two Y Sal TRPM8 cDNAs exist using the following primers.

Y Sal genome F: CTGACTTGGAGGACGCCATGTTCTTG

Y Sal genome R: TCCCTCTTGTTCTGCAGGAC

The resultant fragment was cloned and the nucleotide sequences of seven clones were determined. They were all identical.

For *Xenopus* oocyte expression, each urodelan TRPM8 cDNA (Ax, accession number: LC895907; IR newt, accession number: LC895908; Y Sal, accession number: LC895909 (long), LC895910 (short); JC Sal, accession number: LC895911) was cloned into pGEMHE, which included the 5′ and 3′ noncoding sequence of the Xenopus β‐globin gene [23]. Chimeras were generated by overlapping PCR, and all constructs were verified by sequencing.

### Oocyte electrophysiology

An outline of the methods used for the electrophysiological experiments using *Xenopus* oocytes was described previously [24]. After injecting 50 nL of TRPM8 cRNA (0.1 μg/μl), oocytes were incubated in frog Ringer solution at 17 °C for 3–4 days. Ionic currents were recorded by the two‐electrode voltage clamp method. The recording bath solution contained 96 mm NaCl, 2 mm KCl, 3 mm MgCl_2_, and 5 mm HEPES/NaOH (pH 7.4). Oocytes were voltage‐clamped at −20 mV. The current data were obtained using 400 ms step pulse from −80 mV for 100 ms to + 40 mV for 100 ms and applied every 1 s or 200 ms voltage ramps from −100 mV to + 100 mV applied every 2 s. For cold stimulation, the bath solution was heated to 35 °C with an in‐line heater controller (CL‐100; Warner Instruments, Holliston, MA, USA), and then the ice‐cold bath solution was applied to 15 °C by perfusion. Outward currents were analyzed. When comparing the cold‐induced responses, they were converted into cold responses per menthol response to compensate for differences in the expression levels of the channels. Temperature coefficient Q10 was determined and used to characterize the temperature dependence of the ionic currents as previously described [25].

### Statistical analyses

The values and error bars shown in the figures indicate the mean and standard errors. The statistical significance for the difference of two groups was determined by a Student's two‐sided unpaired *t*‐test, and *P* values were indicated in italics. For other multiple comparisons, statistical significance for the difference was determined by one‐way ANOVA with Tukey–Kramer multiple comparison method. In some cases, statistical significance for the differences with the control group was determined by Dunnett's test. Significant differences were indicated by * (*P* < 0.05), ** (*P* < 0.01), and *** (*P* < 0.001).

### *In silico* analysis of TRPM8 protein

Structural model of human TRPM8 protein was generated by AlphaFold2 [26].

## Results

### Behavioral response to cold temperature of Yamato salamander

**Fig. 1:**
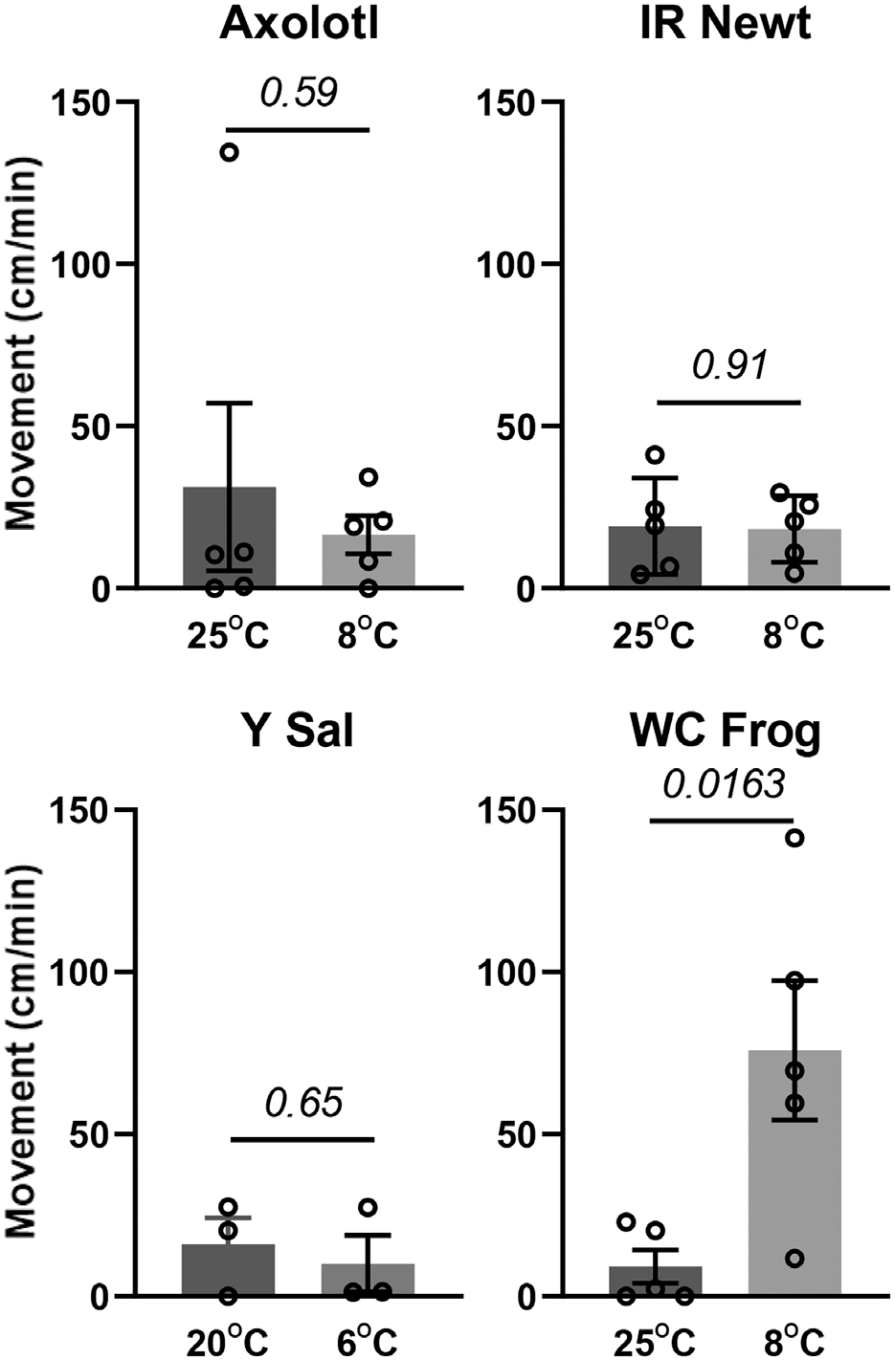
Behavioral responses to cold stimulation in tailed amphibians. Axolotls (ax, *Ambystoma mexicanum*), Iberian ribbed newt (IR Newt, *Pleurodeles waltl*), Yamato salamander (Y Sal, *Hynobius vandenburghi*), and western clawed frog (WC frog, *Xenopus tropicalis*) were used. Each amphibian was placed into a plastic round case beaker with chlorine‐free tap water at room or cold temperatures. We monitored the locomotor activity of the animals using a video camera. After placing into the tap water, cold‐induced movements per 1 s were measured over 0–60 s and the mean movement (cm) per 1 min was calculated. Each data point (individual symbol) represents a single biological replicate, and the mean ± S.E. was indicated. The statistical significance for the difference of two groups was determined by a Student's two‐sided unpaired *t*‐test and *P* values were indicated in italics (*n* = 3 for Y Sal; 5 for ax, IR newt, and WC frog).

We previously examined noxious effects of mild heat on behavior of tailed amphibians [9]. Here, we investigated effects of cold temperature on behavior of Axolotl (Ax, *Ambystoma mexicanum*), Iberian ribbed newt (IR Newt, *Pleurodeles waltl*), and Yamato salamander (Y Sal, *Hynobius vandenburghi*), and compared these effects with that of western clawed frog (WC frog, *Xenopus tropicalis*). As indicated in Fig. 1, we observed that movements of WC frog were induced significantly by cold temperature at 8 °C (*P* = 0.0163, *n* = 5), whereas those of all three tailed amphibians were not induced by cold temperature at 8 °C or 6 °C (Ax: *P* = 0.59, *n* = 5; IR Newt: *P* = 0.91, *n* = 5; Y Sal: *P* = 0.65, *n* = 3). Thus, we observed that tailed amphibians do not show locomotion to avoid cold temperature, which is apparently different from the cold‐sensing property of WC frog.

### Yamato salamander expresses two splice variants of TRPM8

**Fig. 2:**
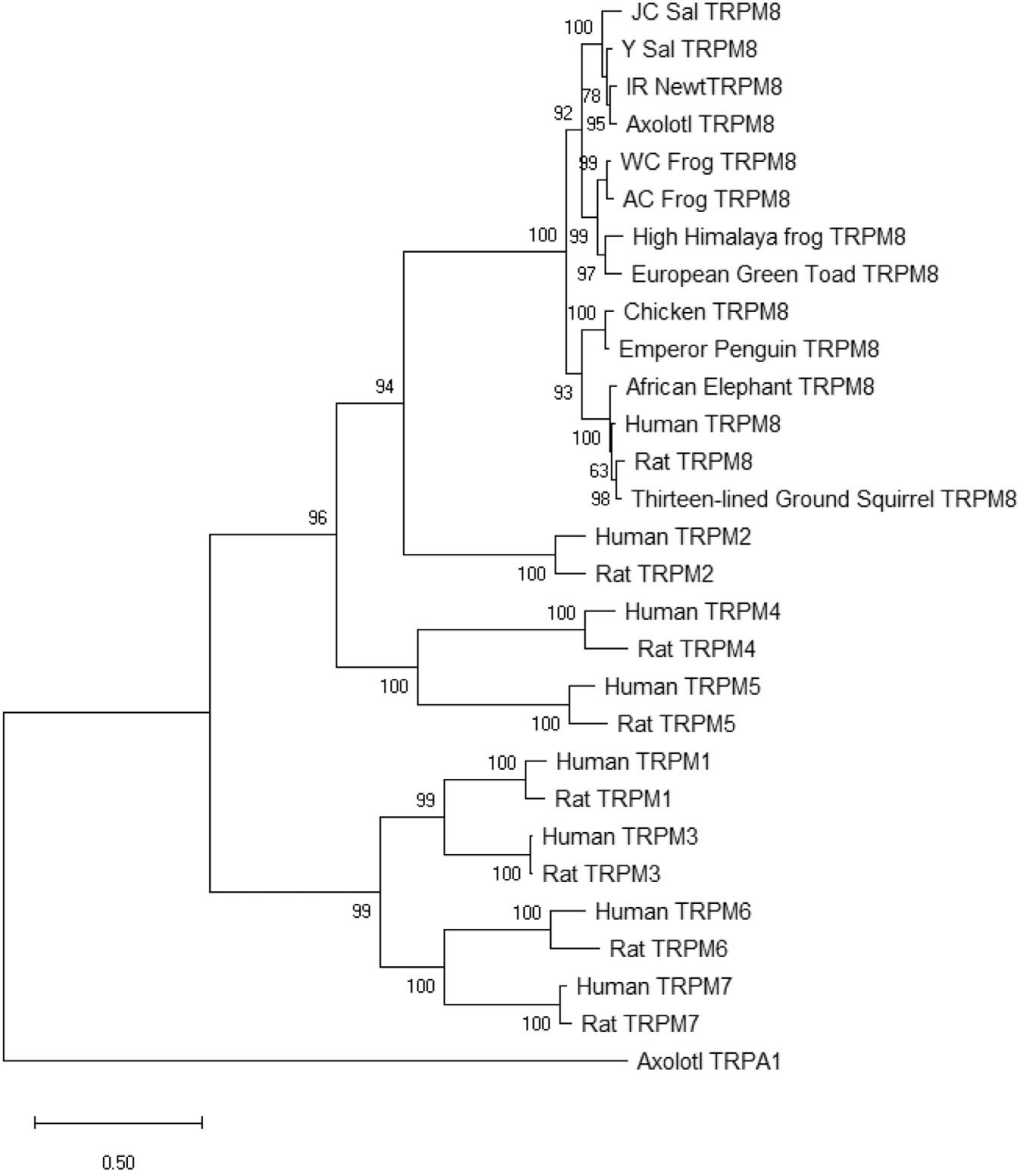
Phylogenetic tree of TRPVM8. Amino acid sequences of MHR1‐transmembranes (TM1–TM6) of TRPM8 from tailed amphibians, frogs, birds, mammals, and TRPM1–7 from mammals, and axTRPA1 were used for a maximum likelihood analysis with MEGA X (https://www.megasoftware.net).

We focused on TRPM8 from tailed amphibians, which do not show significantly elevated locomotion to cold temperature. We first performed isolation and characterization of TRPM8 cDNA of Y Sal. For Y Sal, in winter, its breeding behavior begins in water at nearly 4 °C. We expected that Y Sal TRPM8 may show unique properties. By molecular cloning, we obtained two cDNAs encoding Y Sal TRPM8 with different lengths of 15 nucleotides. Comparison of nucleotides and translation showed that differences between two molecules are only these consecutive 15 nucleotides and a single nucleotide change (Fig. S1). To investigate the mechanism by which two types of TRPM8 arise, we determined the partial genomic sequence encoding TRPM8. We amplified genomic sequence surrounding regions where differences between the two molecular species exist and determined the nucleotide sequence. Two alternate 5′ splice sites of intron were found, and the nucleotide at the single nucleotide change position on the genome was C of long‐type TRPM8 (Fig. S2). Therefore, we considered that a single Y Sal TRPM8 gene produces these two molecules and that single nucleotide change is caused by RNA editing (1583C to U). Further, it was revealed that each of these two Y Sal TRPM8 cDNAs encodes a typical TRPM8 molecule composed of four melastatin homology regions (MHRs) and six transmembrane domains (TMDs). The five amino acid insertion and the single amino acid change were found to be located between the 3rd and 4th MHR. Thus, Y Sal expresses two splice variants of TRPM8 long and short (L and S). We further isolated TRPM8 cDNAs for Ax, IR newt, and JC Sal. We could not find any splice variant for TRPM8 of Ax, IR newt, and JC Sal. Homology comparison indicated that identities on amino acid of Y Sal TRPM8 long to TRPM8s from various animals ranged from 75% (rat) to 92% (tailed amphibians). Phylogenetic analysis using the deduced sequence of amino acids of TRPM8s from tailed amphibians showed one clade, which is diverse from the clade of frog TRPM8s. Then, the clade of amphibian TRPM8s (tailed amphibians and frogs) clustered with the clade of other vertebrate TRPM8s (birds and mammals) (Fig. 2).

### Yamato salamander TRPM8 has diminished cold sensitivity

**Fig. 3:**
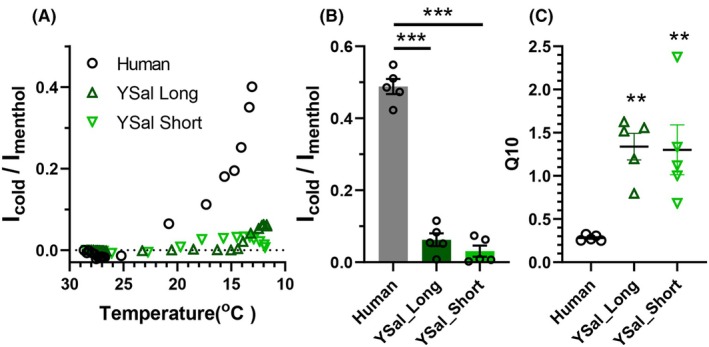
Cold activation property of TRPM8 from Yamato salamander. (A) Response to cold of the TRPM8 from Y Sal. Y Sal TRPM8 was expressed in *Xenopus* oocytes and the cold‐induced response was examined by the two‐electrode voltage clamp method as described in the Methods section. The representative cold‐induced currents standardized by the menthol (0.4 mm for human, 3 mm for Y Sal Long, and 5 mm for Y Sal Short)‐induced currents at + 40 mV are indicated. (B) Average currents for cold stimulation at 13 °C standardized by the menthol‐induced currents at + 40 mV of each TRPM8 were compared (*n* = 5). Each data point (individual symbol) represents a single biological replicate, and the mean ± S.E. was indicated. Statistically significant differences were determined by one‐way ANOVA with Tukey–Kramer multiple comparison method. Significant differences were indicated by *** (*P* < 0.001). (C) Average temperature coefficient (Q_10_) was compared (*n* = 5). Q_10_ value was determined for steep component of the cold‐evoked currents between 26 °C and 16 °C. Each data point (individual symbol) represents a single biological replicate, and the mean ± S.E. was indicated. Statistical significance for the differences with human TRPM8 group was determined by Dunnett's test and indicated by ** (*P* < 0.01).

We analyzed the ligand sensitivity of TRPM8 long and short from Y Sal using menthol, a well‐recognized cooling reagent, which activate known TRPM8 ion channels by two‐electrode voltage clamp in *Xenopus* oocytes in comparison with human TRPM8. All TRPM8 showed menthol‐evoked currents in a dose‐dependent manner. The estimated EC50s for menthol were 0.36 ± 0.06 mm for human TRPM8, 8.27 ± 3.13 mm for Y Sal TRPM8S, and 3.11 ± 0.93 mm for Y Sal TRPM8L, respectively (Fig. S3). We next measured cold activation compared with menthol activation. As expected, human TRPM8 was gradually activated in response to cooling solution from 25 °C to 20 °C and robust activated by further cooling. In contrast, both Y Sal TRPM8s showed only slightly increased activity (Fig. 3). Thus, cold response of Y Sal TRPM8s were significantly reduced compared with that of human TRPM8 (*n* = 5, currents: *P* < 0.001; Q_10_: *P* < 0.01). The diminished cold sensitivity of Y Sal TRPM8s was consistent with the apparent cold tolerance of Y Sal observed by the behavior analysis (Fig. 1).

### Melastatin homology domain 3 plays an essential role in diminishing cold sensitivity of Y Sal TRPM8

**Fig. 4:**
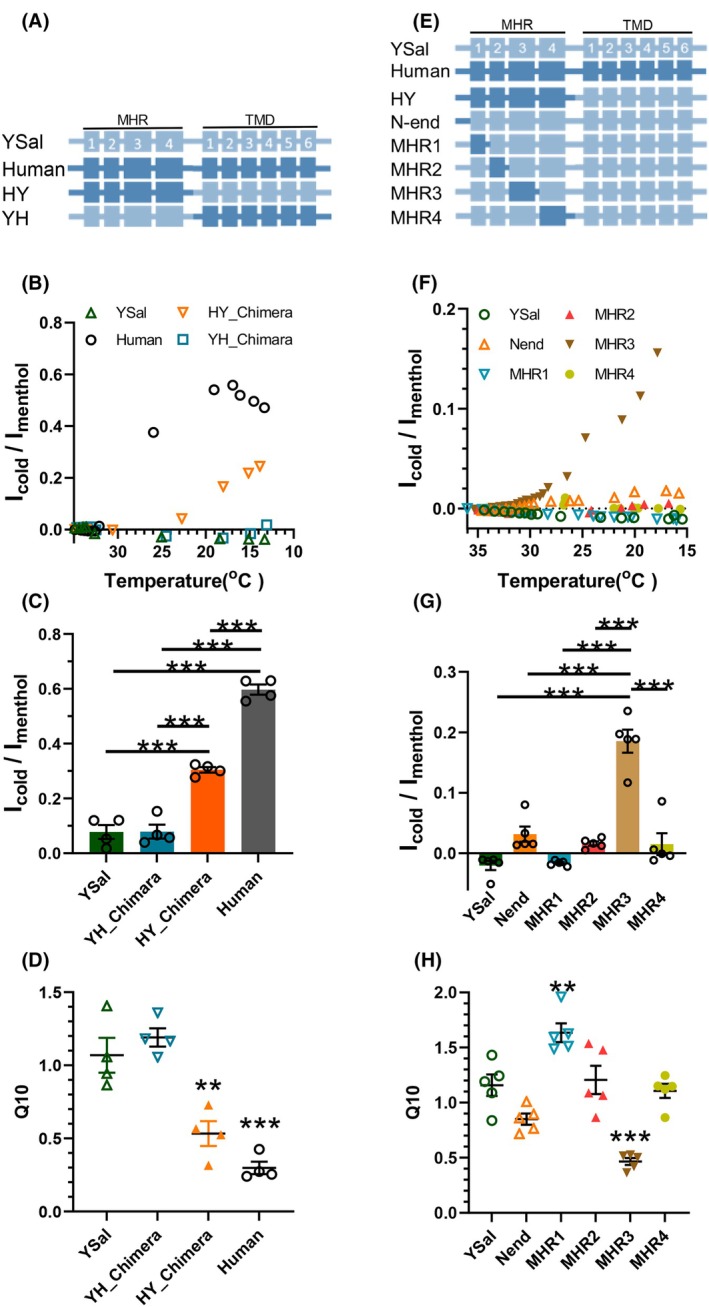
Melastatin homology region 3 determines the diminished cold response of Yamato salamander. (A) schematic representation of Y Sal TRPM8 chimeras. Chimeric channels were generated between Y Sal (light blue) and human TRPM8 (blue) on the basis of the location of melastatin homology regions (MHR1–4) and transmembrane domains (TMD1–6). (B) Each of two chimeras (HY and YH), Y Sal, or human TRPM8 was expressed in *Xenopus* oocytes and the cold‐induced currents were examined by the two‐electrode voltage clamp method. The representative cold‐induced currents standardized by the menthol (0.4 mm for human, 20 mm for others)‐induced currents at + 60 mV are indicated. (C) Average current for cold stimulation at 14 °C standardized by the 20 mm menthol‐induced currents at + 60 mV of each chimera in oocytes were compared (*n* = 4). Each data point (individual symbol) represents a single biological replicate, and the mean ± S.E. was indicated. Statistically significant differences were determined by one‐way ANOVA with Tukey–Kramer multiple comparison method, and significant differences were indicated by *** (*P* < 0.001). (D) Average Q_10_ was compared (*n* = 4). Q_10_ value for human was determined for steep component of the cold‐evoked currents between 30 °C and 20 °C and Q_10_ values for others were determined between 26 °Cand 16 °C. Each data point (individual symbol) represents a single biological replicate, and the mean ± S.E. was indicated. Statistical significance for the differences with YSal TRPM8 group was determined by Dunnett's test and indicated by ** (*P* < 0.01) or *** (*P* < 0.001). (E) Schematic representation of Y Sal TRPM8 chimeras. (F) Each of five chimeras (N‐end, MHR1‐4) or Y Sal TRPM8 was expressed in *Xenopus* oocytes and the cold‐induced currents were examined by the two‐electrode voltage clamp method. The representative cold‐induced currents standardized by the menthol (20 mm)‐induced currents at + 60 mV are indicated. (G) Average current for cold stimulation at 16 °C standardized by the 20 mm menthol‐induced currents at + 60 mV of each chimera in oocytes were compared (*n* = 5). Each data point (individual symbol) represents a single biological replicate, and the mean ± S.E. was indicated. Statistically significant differences were determined by one‐way ANOVA with Tukey–Kramer multiple comparison method, and significant differences were indicated by *** (*P* < 0.001). (H) Average Q_10_ was compared (*n* = 5). Q_10_ value was determined for steep component of the cold‐evoked currents between 26 °C and 16 °C. Each data point (individual symbol) represents a single biological replicate, and the mean ± S.E. was indicated. Statistical significance for the differences with YSal TRPM8 group was determined by Dunnett's test and indicated by ** (*P* < 0.01) or *** (*P* < 0.001).

Functional analysis indicated that both Y Sal TRPM8s have quite similar properties, and we further characterized the long form. To determine structural elements that confer the diminished cold sensitivity in Y Sal TRPM8L, we made chimeric channels with the cold‐sensitive human ortholog and examined them by two‐electrode voltage clamp using *Xenopus* oocytes. First, the substitution of N‐terminal MHRs or C‐terminal TMDs in Y Sal TRPM8L with homologous domains from human TRPM8 (HY and YH) was performed. While YH chimera showed diminished response to the cold temperature, a significant activation of HY chimera upon cooling was observed (*n* = 4, currents: *P* < 0.001; Q_10_: *P* < 0.01, Fig. 4A–D). Results demonstrated important roles of N‐terminal MHRs in the diminished cold sensitivity in Y Sal TRPM8L.

Next, we examined the roles of each Melastatin homology region (MHR1‐4) of Y Sal TRPM8L in reducing cold sensitivity by constructing five chimeras (Fig. 4E). The N‐terminal end part, MHR1, MHR2, MHR3, or MHR4 of Y Sal TRPM8L, was exchanged with the corresponding part of human TRPM8, and the cold‐response was electrophysiologically examined (Fig. 4E–H). Results indicated that replacing the Y Sal MHR3 with human TRPM8 introduced discernible cold‐induced activation (*n* = 5, currents: *P* < 0.001; Q_10_: *P* < 0.001), and this region is suggested to play an important role in dictating the reduced cold sensitivity of Y Sal TRPM8L.

**Fig. 5:**
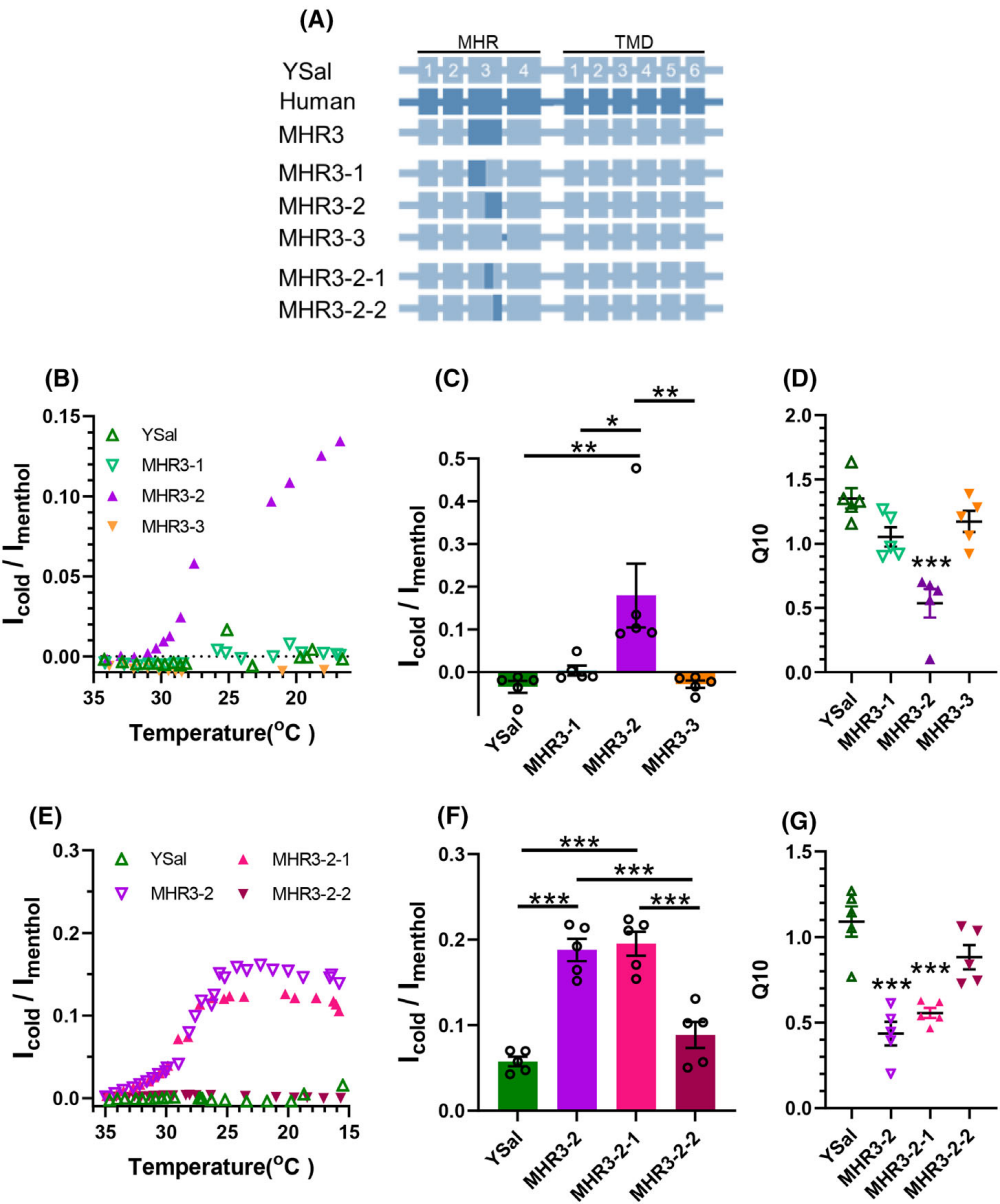
Essential role of 48 amino acids in MHR3 in the cold‐insensitivity of Yamato salamander TRPM8. (A) schematic representation of Y Sal TRPM8 chimeras. (B) Each of chimeras (MHR3‐1, ‐2, ‐3) or Y Sal TRPM8 was expressed in *Xenopus* oocytes, and the cold‐induced currents were examined by the two‐electrode voltage clamp method as indicated. The representative cold‐induced currents standardized by the menthol (20 mm)‐induced currents at + 60 mV are indicated. (C) Average current for cold stimulation at 16.2 °C standardized by the 20 mm menthol‐induced currents at + 60 mV of each chimera in oocytes were compared (*n* = 5). Each data point (individual symbol) represents a single biological replicate, and the mean ± S.E. was indicated. Statistically significant differences were determined by one‐way ANOVA with Tukey–Kramer multiple comparison method, and significant differences were indicated by * (*P* < 0.05) and ** (*P* < 0.01). (D) Average Q_10_ was compared (*n* = 5). Q_10_ value was determined for steep component of the cold‐evoked currents between 30 °C and 20 °C. Each data point (individual symbol) represents a single biological replicate, and the mean ± S.E. was indicated. Statistical significance for the differences with YSal TRPM8 group was determined by Dunnett's test and indicated by *** (*P* < 0.001). (E) Each of chimeras (MHR3‐2, 3‐2‐1, 3‐2‐2) or Y Sal TRPM8 was expressed in *Xenopus* oocytes and the cold‐induced currents were examined by the two‐electrode voltage clamp method. The representative cold‐induced currents standardized by the menthol (20 mm)‐induced currents at + 60 mV are indicated. (F) Average current for cold stimulation at 17 °C standardized by the 20 mm menthol‐induced currents at + 60 mV of each chimera in oocytes were compared (*n* = 5). Each data point (individual symbol) represents a single biological replicate, and the mean ± S.E. was indicated. Statistically significant differences were determined by one‐way ANOVA with Tukey–Kramer multiple comparison method, and significant differences were indicated by *** (*P* < 0.001). (G) Average Q_10_ was compared (*n* = 5). Q_10_ value was determined for steep component of the cold‐evoked currents between 30 °C and 20 °C. Each data point (individual symbol) represents a single biological replicate, and the mean ± S.E. was indicated. Statistical significance for the differences with YSal TRPM8 group was determined by Dunnett's test and indicated by *** (*P* < 0.001).

**Fig. 6:**
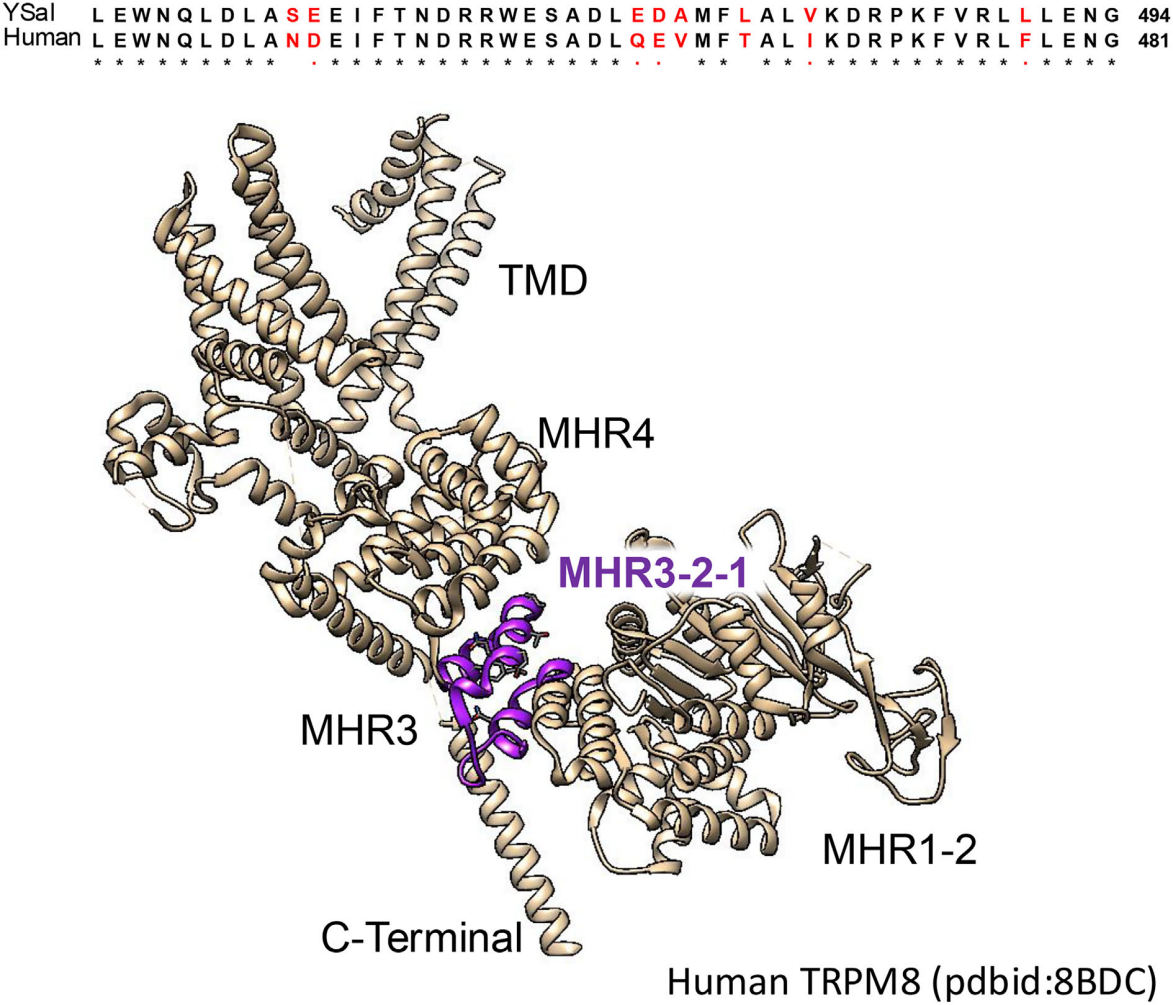
Specific 8 amino acids of MHR3 for Yamato salamander. The amino acid sequences of MHR3‐2‐1 of Y Sal and human TRPM8 are indicated. Human TRPM8 (AlphaFold2 model) is further shown. Ribbon diagram of a human TRPM8 monomer denoting specific domains.

To narrow down the responsible region for the reduced cold‐sensitivity of Y Sal TRPM8L, its MHR3 was divided into three parts and each part was exchanged with the corresponding part of human TRPM8 (Fig. 5A). The middle part of MHR3 (HMR3‐2) of human TRPM8 allows Y Sal TRPM8L to significantly respond to cold temperature (*n* = 5, currents: *P* < 0.01; Q_10_: *P* < 0.001). Exchange of the other two parts of MHR3 showed almost no effect on the cold‐insensitivity of Y Sal TRPM8L (Fig. 5B–D). Then, MHR3‐2 was divided into two parts (MHR3‐2‐1, MHR3‐2‐2) and each part was exchanged with the corresponding part of human TRPM8 (Fig. 5A). The MHR3‐2‐1 of human TRPM8 was able to confer cold sensitivity on Y Sal TRPM8L (*n* = 5, currents: *P* < 0.001; Q_10_: *P* < 0.001), but replacing MHR3‐2‐2 on Y Sal TRPM8L with the human ortholog did not show the activation by cold temperature (Fig. 5E–G). As indicated in Fig. 6, MHR3‐2‐1 contains well‐conserved 48 amino acids and only eight amino acids (AAs) are specific for Y Sal TRPM8L. Therefore, it was strongly suggested that these eight AAs must play important roles in decreasing the cold sensitivity of Y Sal TRPM8L. Whether all eight AAs are necessary or not, however, remains unclear at this point, and further point mutation experiments are required in the future.

### TRPM8s from tailed amphibians commonly have the diminished cold sensitivity

**Fig. 7:**
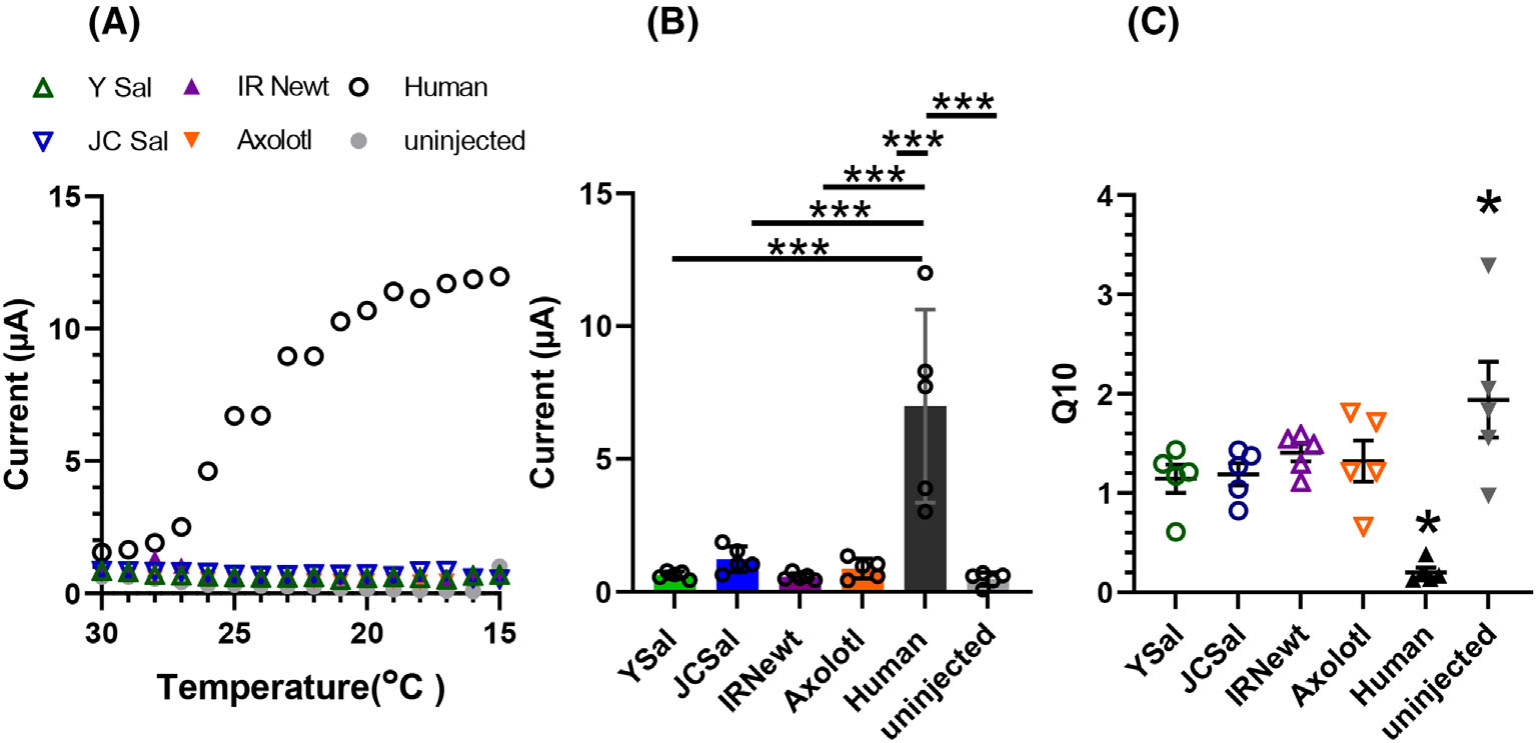
Cold‐induced response of TRPM8 from four‐tailed amphibians. (A) JC Sal, Y Sal, IR Newt, Ax, or human TRPM8 was expressed in *Xenopus* oocytes and the cold‐induced currents were examined by the two‐electrode voltage clamp method. The representative cold‐induced currents at + 40 mV are indicated. (B) Average current for cold stimulation at 15 °C of each TRPM8 in oocytes were compared (*n* = 5). Each data point (individual symbol) represents a single biological replicate, and the mean ± S.E. was indicated. Statistically significant differences were determined by one‐way ANOVA with Tukey–Kramer multiple comparison method, and significant differences were indicated by *** (*P* < 0.001). (C) Average Q_10_ was compared (*n* = 5). Q_10_ value was determined for steep component of the cold‐evoked currents between 30 °C and 20 °C. Each data point (individual symbol) represents a single biological replicate, and the mean ± S.E. was indicated. Statistical significance for the differences with YSal TRPM8 group was determined by Dunnett's test and indicated by * (*P* < 0.05).

**Fig. 8:**
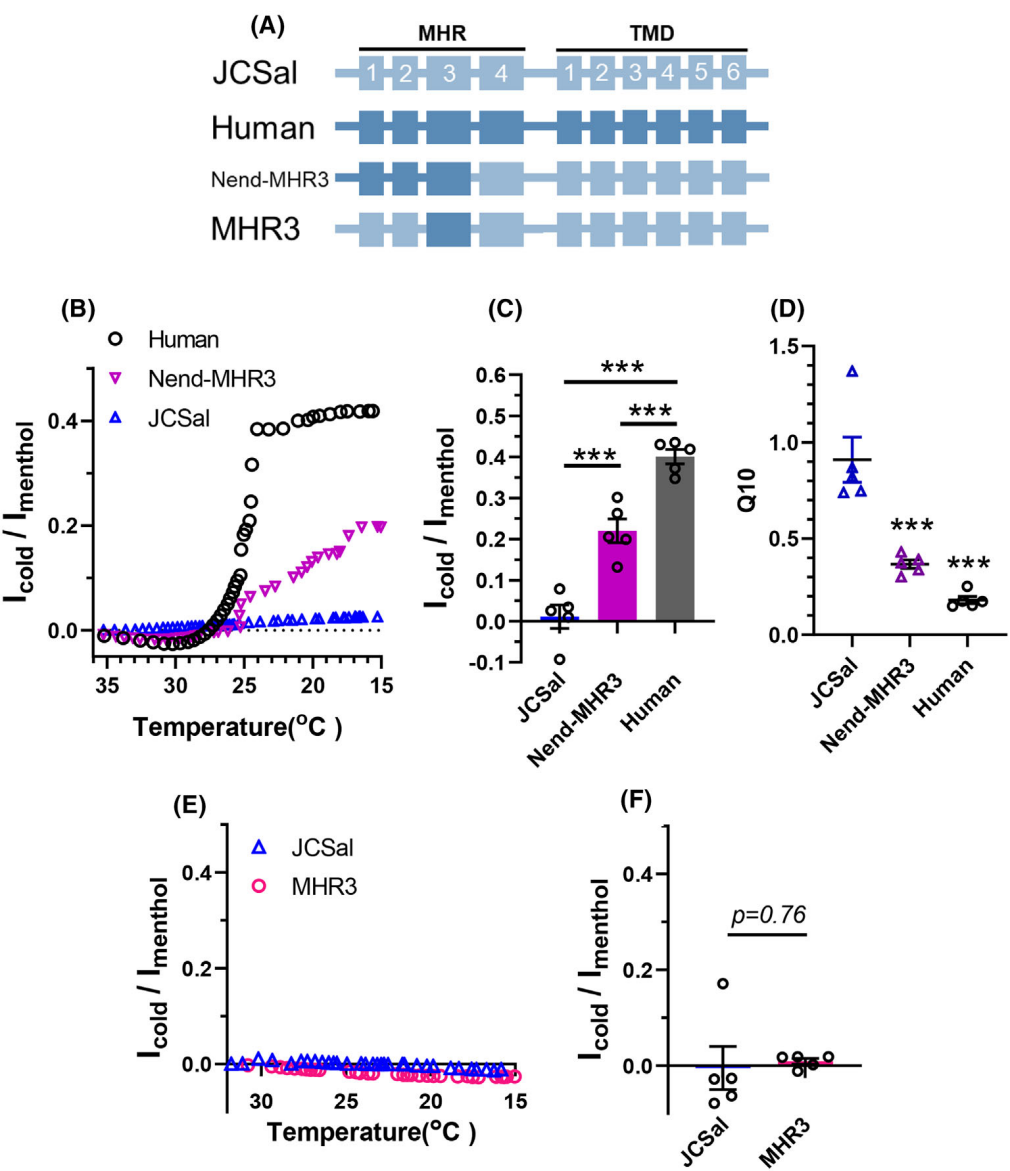
Role of MHR3 in the cold‐insensitivity of Japanese clawed salamander TRPM8. (A) schematic representation of JC Sal TRPM8 chimeras. (B) JC Sal, human, or chimera (Nend‐MHR3) TRPM8 was expressed in *Xenopus* oocytes and the cold‐induced currents were examined by the two‐electrode voltage clamp method. The representative cold‐induced currents standardized by the menthol (0.4 mm for human, 2 mm for JC Sal and chimera)‐induced currents at + 80 mV are indicated. (C) Average current for cold stimulation at 16 °C standardized by the menthol‐induced currents at + 80 mV of JC Sal, human, or chimera TRPM8 in oocytes were compared (*n* = 5). Each data point (individual symbol) represents a single biological replicate, and the mean ± S.E. was indicated. Statistically significant differences were determined by one‐way ANOVA with Tukey–Kramer multiple comparison method, and significant differences were indicated by *** (*P* < 0.001). (D) Average Q_10_ was compared (*n* = 5). Q_10_ value for human was determined for steep component of the cold‐evoked currents between 30 °C and 20 °C and Q_10_ values for others were determined between 26 °C and 16 °C. Each data point (individual symbol) represents a single biological replicate, and the mean ± S.E. was indicated. Statistical significance for the differences with JCSal TRPM8 group was determined by Dunnett's test and indicated by *** (*P* < 0.001). (E) Chimera (MHR3) or JC Sal TRPM8 was expressed in *Xenopus* oocytes and the cold‐induced currents were examined by the two‐electrode voltage clamp. The representative cold‐induced currents standardized by the menthol (2 mm)‐induced currents at + 80 mV are indicated. (F) Average current for cold stimulation at 16 °C standardized by the 2 mm menthol‐induced currents at + 80 mV of Chimera (MHR3) or JC Sal TRPM8 in oocytes were compared (*n* = 5). Each data point (individual symbol) represents a single biological replicate, and the mean ± S.E. was indicated. The statistical significance for the difference of two groups was determined by a Student's two‐sided unpaired *t*‐test, and *P* value was indicated in italics.

As shown in Fig. 1, we investigated the effects of cold temperature on behavior of Ax, IR Newt, and Y Sal, and compared with that of WC frog. Movements of WC frog were induced elevated by cold temperature, but all three tailed amphibians did not show the cold induced response. When TRPM8L and TRPM8S were cloned from Y Sal and the cold response was studied, both of Y Sal TRPM8s were shown to have significantly decreased cold sensitivity. These characteristics may contribute to the cold tolerance observed by behavior experiment. To determine whether the diminished cold sensitivity is a common feature of TRPM8 of tailed amphibians, we further performed functional analyses of TRPM8s from Ax, IR newt, and JC Sal. In addition to Y Sal TRPM8s, all of these three TRPM8 of Ax, IR Newt, and JC Sal were activated by menthol (*P* < 0.01, *n* = 5, Fig. S3). When the cold response of each TRPM8 of tailed amphibians was investigated, none of them responded at all upon cooling to 15 °C (*n* = 5, currents: *P* < 0.001; Q_10_: *P* < 0.05 Fig. 7). Results clearly demonstrated that TRPM8s from tailed amphibians commonly have diminished cold sensitivity, while frog TRPM8 was known to be activated by cold temperature [15, 19]. In case of Y Sal TRPM8L, we showed an important role of MHR3 in diminishing cold sensitivity. To examine whether the decreased cold sensitivity of tailed amphibian TRPM8 might be similarly determined, we studied the cold sensitivity of the chimeras between JC Sal and human TRPM8s. First, the N‐terminal fragment containing 3MHRs (MHR1‐3) of JC Sal TRPM8 was replaced with the corresponding part of human TRPM8 (Nend‐MHR3). As shown in Fig. 8B–D, this Nend‐MHR3 chimera was able to respond to the cold stimulation (*n* = 5, currents: *P* < 0.001; Q_10_: *P* < 0.001), suggesting that structural elements conferring the diminished cold sensitivity of JC Sal TRPM8 may be present in the MHR1‐3. Then, based on previous experiments on Y Sal TRPM8, we investigated the involvement of MHR3 in decreasing the cold sensitivity of JC Sal TRPM8. Although the sequences of MHR3 are well‐conserved between Y Sal and JC Sal TRPM8, the exchange of MHR3 of JC Sal TRPM8 with human TRPM8 could not provide the cold sensitivity to JC Sal TRPM8 (*P* = 0.76, *n* = 5, Fig. 8E,F). Results demonstrated that elements conferring the diminished cold sensitivity in JC Sal TRPM8 may also exist in the region of the N terminus other than MHR3. Further chimeric analysis is required to determine which region of JC Sal TRPM8 is also involved in the loss of cold sensitivity in the future, but our results suggested the possibility that the diminished cold sensitivity of TRPM8 of tailed amphibians such as JC Sal might be acquired by the common ancestral and subsequent species‐specific mutations that further locked the channel in a cold‐insensitive state.

## Discussion

In cold transduction of animals, TRPM8 is known to play important roles [10, 11, 12, 13, 14]. Activation of rat TRPM8 is observed by cold stimulation from 24 °C, but cold sensitivity of TRPM8 is tuned during evolution in several examined vertebrate species [15, 16, 17, 18, 19]. In this study, we focused on tailed amphibians, which prefer cooler environments, studied behavior response to cold temperature, and characterized their TRPM8 function. We observed that tailed amphibians do not show escaping behavior to avoid cold temperature, while frogs avoided cold temperature. We first isolated TRPM8 cDNAs from Yamato salamander (Y Sal, *Hynobius vandenburghi*), and its TRPM8s showed significantly diminished cold response. Further, detailed analysis using chimera channels of Y Sal and human TRPM8 indicated that the MHR3 of human TRPM8 could provide the cold sensitivity to Y Sal TRPM8L, and eight amino acids located in MHR3 play essential roles in decreasing the cold sensitivity of Y Sal TRPM8L. To determine the relative importance of each of the eight amino acids, further detailed studies such as point mutation analysis will be necessary. Then, examination of temperature‐response profiles of TRPM8 from three other tailed amphibians, axolotl (Ax, *Ambystoma mexicanum*), Iberian ribbed newt (IR Newt, *Pleurodeles waltl*), and Japanese clawed salamander (JC Sal, *Onychodactylus japonicus*), demonstrated that tailed amphibian TRPM8s commonly have diminished cold sensitivity. Exchange of MHR3 of JC Sal TRPM8 with human TRPM8, however, could not acquire the cold response. The eight AAs in MHR3 playing important roles in the cold insensitivity of Y Sal TRPM8 are completely conserved among four tailed amphibians. Therefore, the region of the N terminus other than MHR3 further must contribute to the diminished cold sensitivity in JC Sal TRPM8. These observations suggested that each tailed amphibian has developed the cold tolerance by decreasing the cold response of TRPM8 through the common MHR3 mutations and further species‐specific additional mutations outside of MHR3.

Study on TRPM8 from ground squirrel (Sq) and Syrian hamster (Ham) revealed that both TRPM8s have diminished cold sensitivity. Chimera analysis with rat TRPM8 demonstrated that the transmembrane core domain mainly contributes to determine the cold sensitivity of Sq and Ham TRPM8s, and the analysis of mutants replacing individual AA identified six residues in the transmembrane core domain of Sq TRPM8 as determinants for its cold insensitivity. Three of them were located between the 5th and 6th transmembrane. However, the six AAs are not conserved between Sq and Ham TRPM8, suggesting that these TRPM8 channels have lost cold sensitivity through nonidentical changes in the transmembranes [16]. Our study on tailed amphibian TRPM8s suggested that the N‐terminal region containing MHRs may determine the cold sensitivity. Indeed, the six AAs in the transmembranes of Sq TRPM8 playing essential roles in reduced cold responses were not found in the corresponding positions in TRPM8 from tailed amphibians.

Further, it has been reported that the AA change in the six transmembrane region causes a decrease in the low‐temperature sensitivity of TRPM8 of another animal species. The cold response of TRPM8 of the penguin, well known for exhibiting remarkable tolerance to cold, was examined and the cooling activation of the penguin's TRPM8 only induced a minute current level. The chimera and point mutation analysis demonstrated that the 919 AA (Y instead of V) in the pore domain (PD) of the transmembrane core domain is crucial for the decreased cold activation of the penguin's TRPM8 [18]. This AA(Y) is conserved among TRPM8s of tailed amphibians, but our results suggested that the N‐terminal region containing MHRs may contribute to the diminished cold sensitivity of tailed amphibian TRPM8s rather than the transmembrane domain containing Y919.

In 2022, it has been reported that TRPM8s from lungfish, caecilian, and sea turtle do not show activation upon cooling. In this report, MHR1‐3 were considered as a functionally combined domain for TRPM8 cold sensitivity to alter the threshold temperature. For the robustness of the cold response of TRPM8, however, the regulatory mechanism of the PD was suggested to be necessary [19]. Here, we insisted that tailed amphibians have independently acquired the decreased cold sensitivity of TRPM8 by obtaining the mutations in MHR3 and other parts. Thus, in the process of cold adaptation or tolerance to cold and also in the evolutionary process, each animal must have obtained the functional changes in cold sensor, TRPM8 by altering the properties of the common regions (in the N terminus and/or the transmembranes) required to determine the sensitivity to match species‐specific habitat temperature.

So how does the site in MHR3 of Y Sal TRPM8 we found that alters cold sensitivity suppress the responsiveness of TRPM8 to cold temperatures? By incorporation of a fluorescence unnatural amino acid 3‐(6‐acetylnaphthalen‐2‐ylamino)‐2‐aminopropanoic acid (ANAP) into MHR1‐3, MHR4 and pre‐S1 domains of Xt TRPM8 and analyzing the shifts in emission peak of ANAP, the conformational change of MHR1‐3 during cold activation was reported. In particular, the eight AAs in MHR3 of Y Sal TRPM8, which we found to be important in reducing cold sensitivity, were located between L453 and L537 of MHR3 of Xt TRPM8 that were shown to move in the cold‐induced activation [19]. Therefore, these AAs might be involved in ease of structure changes induced by cold stimuli. Indeed, these AAs are completely conserved among TRPM8s of four‐tailed amphibians and all of these TRPM8s showed no cold response. Our finding that a short region of MHR3 determines the low‐temperature sensitivity of TRPM8 is the first finding of its kind. However, how these amino acids are involved in conformational changes and how they relate to the cold‐induced opening of TRPM8 requires further study, including more detailed conformational analysis.

## Conflicts of interest

The authors declare no conflicts of interest.

## Author contributions

SH and OS conducted the main experiments. TS and DK performed the majority of experiments including cloning, mutagenesis, electrophysiological, and animal assays. OS and SH prepared the manuscript. OS conceived and supervised the project.

## Supporting information


**Fig. S1.** Sequences of amino acids of Yamato salamander TRPM8s.

**Fig. S2.** Partial sequence of Yamato salamander TRPM8 gene.

**Fig. S3.** Menthol sensitivity of tailed amphibian TRPM8s.

## Data Availability

The data that support the findings of this study are available from the corresponding author (o_saito@nagahama-i-bio.ac.jp or s_hori@nagahama-i-bio.ac.jp) upon reasonable request.
