# Supplementary figures and images for: Decreased cold‐sensing function of the transient receptor potential channel TRPM8 from tailed amphibians
Source: FEBS Open Bio. 2026 Mar 25;16(8):1477–90. doi: 10.1002/2211-5463.70227 (PMC13398745; doi:10.1002/2211-5463.70227)

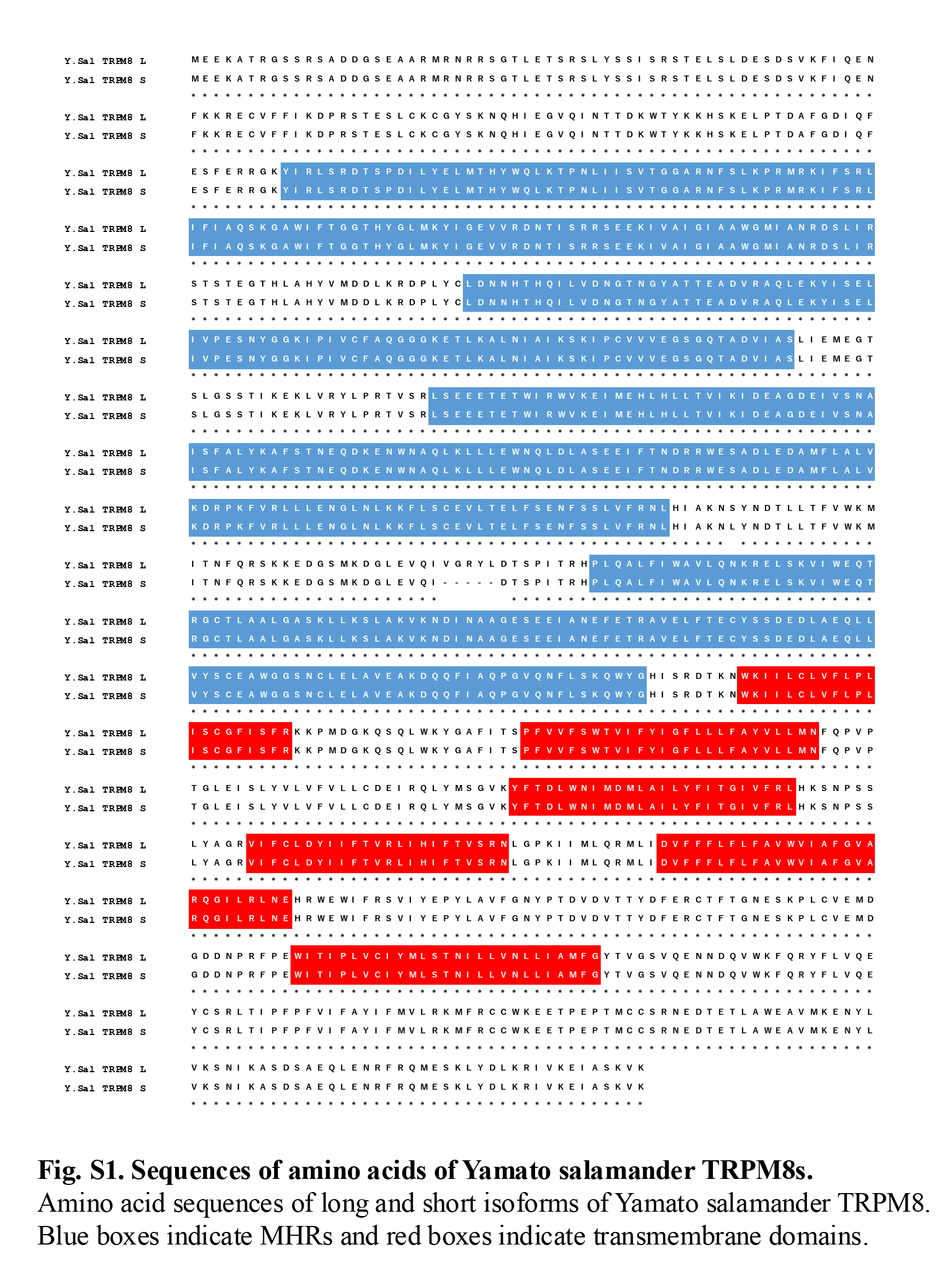

Supplement: Supplementary file 1 — Fig. S1. Sequences of amino acids of Yamato salamander TRPM8s. [file FEB4-16-1477-s001.tif]

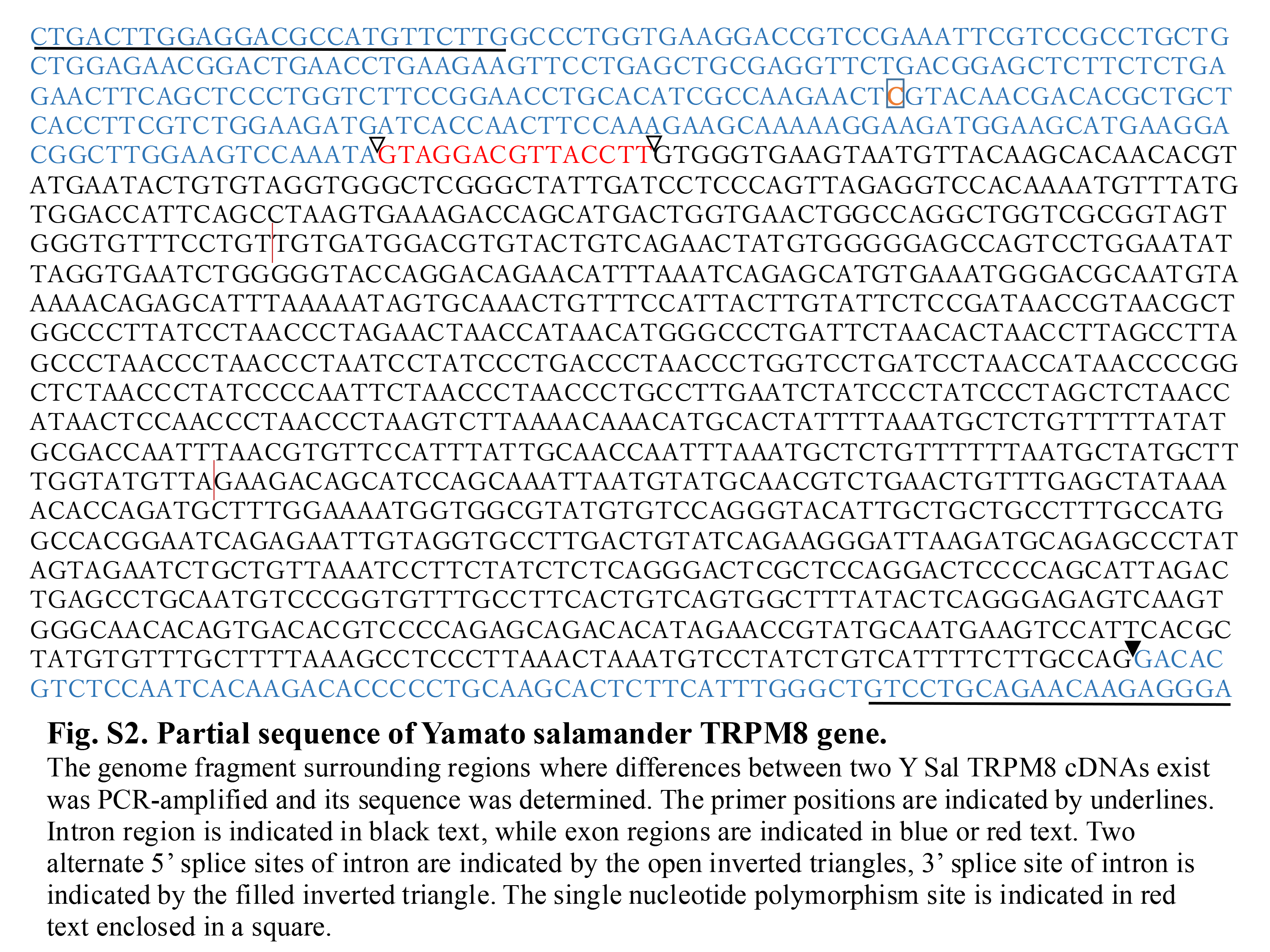

Supplement: Supplementary file 2 — Fig. S2. Partial sequence of Yamato salamander TRPM8 gene. [file FEB4-16-1477-s002.tif]

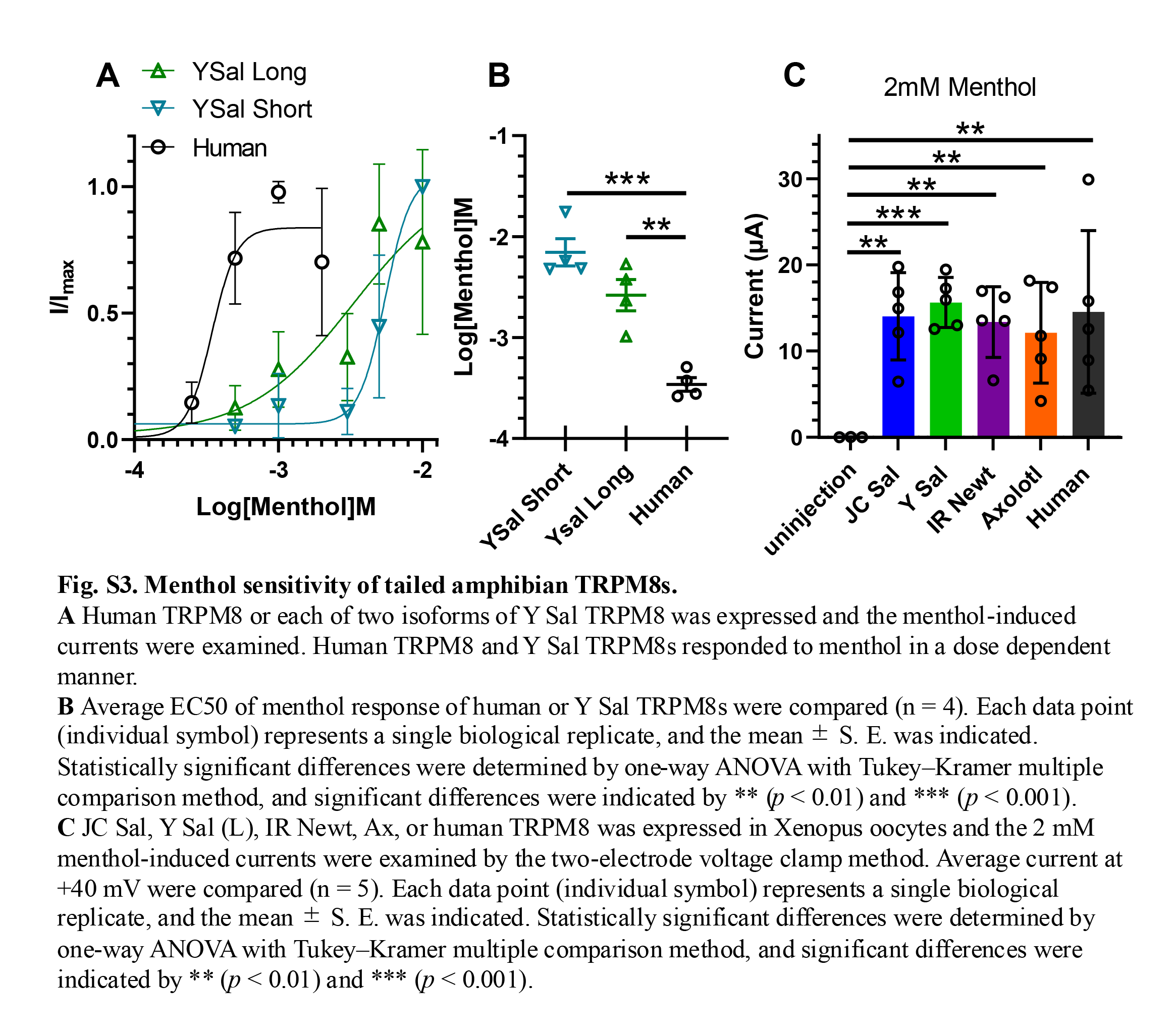

Supplement: Supplementary file 3 — Fig. S3. Menthol sensitivity of tailed amphibian TRPM8s. [file FEB4-16-1477-s003.tif]
